# Supplementary material for: Exploring optimal granularity for extractive summarization of unstructured health records: Analysis of the largest multi-institutional archive of health records in Japan
Source: PLOS Digit Health. 2022 Sep 15;1(9):e0000099. doi: 10.1371/journal.pdig.0000099 (PMC9931252; doi:10.1371/journal.pdig.0000099)
Supplement: Attachment — Submitted filename: Rebuttal letter-20220627.pdf [file pdig.0000099.s001.pdf]

Exploring Optimal Granularity for Extractive Summarization of Unstructured Health Records: Analysis of the Largest Multi-Institutional Archive of Health Records in Japan

Manuscript No. PDIG-D-21-00099

Response to the Editor

> *The study is interesting and explores an important topic. Our reviewers - in particular Reviewer 1 - have raised some points that I think should be addressed prior publication. I would be grateful if you could submit a new version of the paper after responding to these points.*

We thank you for the thoughtful and constructive feedback provided by the reviewers. Most of the comments and suggestions were understandable, except for a few exceptions, and we revised the manuscript accordingly. We also realized some typographical and technical errors in the medical terms translated from Japanese and corrected them. Additionally, we modified the financial disclosures and competing interest statements as requested by the journal office.

To the best of our knowledge, the manuscript now conforms to the journal requirement and meets your publication criteria. Please let us know if there are any areas where we could improve.

Responses to Reviewers

Reviewer 1

General Comment

> *An insightful presentation of the state of the art for clinical health records and discharge summaries in Japanese hospitals as well as the application of natural language processing (NLP) on said clinical text data. NLP in Japanese, let alone for clinical discharge summaries, presents a unique challenge and we applaud the authors for gaining access to the largest clinical text database and the efforts for conducting this study.*
> *However, there were a few key elements that the paper could be revised in order to showcase and highlight the novelty of the study.*

We appreciate the reviewer's comment and detailed feedback. We addressed all the issues raised by the comment as best as we could in the following rebuttals.

Major concern #1*:*

> *The paper did not describe the unique difficulties in conducting NLP in Japanese, such as the lack of spaces between the characters and words. This is further exacerbated with the lack of open source information to combat this, and including such limitations may further emphasize the quality of this study.*

We agree that the uniqueness of the Japanese NLP should have been highlighted. We have modified the related study section in the revised manuscript accordingly.

Major concern #2

> *The authors do not declare any other NLP related models or research available in Japan, and the study does not list many related studies such as the following which would have been helpful in creating comparisons:*
>
> - *"Preliminary development of a deep learning-based automated primary headache diagnosis model using Japanese natural language processing of medical questionnaire"*
> - *"Predicting Inpatient Falls Using Natural Language Processing of Nursing Records Obtained From Japanese Electronic Medical Records: Case-Control Stud"*
> - *"A clinical specific BERT developed using a huge Japanese clinical text corpus"*

We appreciate the suggestions from the reviewer on the related works. However, the previous two studies were not typical "summarization" studies. These studies obtained "new information" that does not appear in the input. We want to get information from input documents, not predict the future. Therefore, we assume that including them would mislead readers, even though they are a representative work of Japanese medical NLP.

However, we admit that the related work section was somewhat incomplete; thus, we reorganized the section. Regarding the UTH-BERT study suggested by the reviewer, it was cited in the submitted manuscript as "under review." Because the status of the study has changed during the reviewing period of our manuscript, we updated the bibliography information in the revision.

Major concern #3

> *Relatedly to the aforementioned point, due to the lack of related studies, the authors do not mention how different or novel their research stands in comparison to other Japanese or oversea models.*

We rewrote the second paragraph of the related works section and elaborated on the contribution of our study compared with other models.

Major concern #4

> *Furthermore, the authors mention that the results might be applicable only to Japanese clinical settings, yet since no other examples are given, this cannot be generalized and further examples as well as background information and explanation is required to make this claim.*

As described in Section 3.2, we annotated Japanese texts to address Japanese-specific issues. Therefore, our results apply to clinical narratives written in Japanese. New analysis in the target languages would be required to use our method in other languages. We have added detailed explanations regarding these in the discussion section to clarify the point.

Major concern #5

> *The authors mention that their model requires entire documents for training, and that the number of documents in their corpus was too small to be used for the training of the model; they however do not explain why this is the case. Perhaps by including examples from Japan, if not from abroad, would help readers understand their model's novelty.*

The primary reason why larger data sets are better for training models is to improve the robustness of the model to unknown test data. The number of cases in the NHO data is more than 200 times greater than that in our corpus. We have added the explanation in Section 4.3.

Major concern #6

> *The authors mention that in this experiment, they used three contextual units instead of n-gram units to evaluate their impact on the summarization performance and determine which unit performs the best. While this is interesting, the authors once again do not describe why they did this and why this is meaningful.*

Section 3.2 discusses the issue and reason for using three contextual units. Previous studies used sentences and clauses as self-contained linguistic units for extractive summarization. However, this setting does not demonstrate that clauses are the best fit as a unit for extractive summarization. Furthermore, Japanese clauses are often very short at the phrase level compared with English. Therefore, we defined a new self-contained linguistic unit that is longer than a clause and represents the smallest medically meaningful concept.

Major concern #7

> *The authors discuss that this study had used the largest multi-institutional health records archive in Japan and thus it would be worthwhile to validate the results in multiple languages, but if there are actually no other databases, perhaps the authors must first be validating in other Japanese clinical text databases, or other Asian languages that have similar grammatical structures, prior to applying firsthand to other languages that may have more open source resources available.*

The comment covers two distinct topics. First, the description, "*... this study used the largest multi-institutional health records archive…*," asserts the need for domestic generalizability. Second, the description, "*... worthwhile to validate the results in multiple languages,*" suggests that the clinical segment concept can be used in other languages. We have modified the paragraph to make this point clearer.

Major concern #8

The authors raised an interesting point that Japanese hospitals do not use dictation to produce discharge summaries, which could result in frequent copying and pasting from sources to summaries. However, it would be stronger for the authors to provide examples for how this enhances (or does not enhance) the model, as well as further expand on the clinical significance or rationale to dictate or not dictate, and whether there are differences in clinical outcomes or clinical workflow to explore the strengths and weaknesses of the authors' model.

The dictation process may have a positive or negative impact on the generation of discharge summaries. As suggested by the reviewer, this issue might be a key to further research efforts in this problem domain. However, it would require additional experiments to empirically discuss the issue, which is beyond the scope of this study. Therefore, it is left for future study, as we stated in our discussion section.

Major concern #9

Finally (and related to the point above), the authors do not clearly discuss why this study is useful, especially its application to real world clinical settings.

We acknowledge that the detailed rationale for the study may have been missing from the submitted manuscript. Therefore, we modified the related work section in the revision to include the advantage of extractive methods in the clinical narrative summarization. First, the extraction would result in more reliable information. This is an advantageous property for clinical practice, where misinformation is critical. Second, the precise spanning of extraction would be read to avoid the extraction of unnecessary information, keeping processing precision high.

Major concern #10

While the English and Japanese translations available are also interesting, some of the translation seem non-native or slightly incorrect, thus perhaps could use proof-reading and reconsideration (e.g. 髄液糖定量 - glucose determination → cerebrospinal fluid glucose level or glycorrhachia).

We appreciate the comment. This translation required more scrutiny. In the revised manuscript, a domain expert checked the translation again and made necessary corrections.

Reviewer 2

General Comment

> *The study aims to identify the optimal granularity between 3 proposed granularities for performing extractive summary of Japanese clinical texts. The paper is clear and well-written.*

We appreciate the general comments and the detailed feedback. We state the responses to the comments by the reviewer below.

Comment #1

> *Taking into consideration that the exploration of optimal granularity for extractive summary had been previously explored, the innovation of the research, is the choice of the clinical domain. However it is not clear if the results were different from the results reached in other non-clinical domains.*

Understandably, the result might be presented in a more generalized form. However, as elaborated in the methodology section, the analysis has been fine-tuned in the clinical documents, which have various special properties compared to ordinarily texts. For example, shorthand notations are frequently used in clinical narratives, resulting in grammatically incorrect sentences. Furthermore, the clinical segment is defined using medical knowledge and does not correspond to the general domain. Therefore, we assume that the comparison with nonclinical domains may not contribute to the paper quality.

Comment #2

> *The fact that "20-31% of the sentences in discharge summaries were created by copying and pasting", made the authors conclude that a certain amount of content can be automatically generated by extractive summarization.*
> *However since most of the summary needs to be generated by other methods (e.g. abstractive summary), the fact that extractive summarization cannot be used to generate a full summary, raises questions about its use at all in the summarization process of clinical summaries. Would be whether other methods would make any use of the 20% that can be generated by extractive summary?*

This point is mentioned in the introduction but could have been emphasized more in the submitted manuscript. First, it is important to obtain reliable information, especially in clinical practice where misinformation is critical; thus, we analyzed how best we could extract information from the source texts. Second, discharge summaries include clinical facts and physicians' interpretations of the records; thus, they include extractive information, abstractive information, and clinical inference results. The detailed analysis of the summarization process and the composition of the output texts are left for our next study, which is also under review by PLOS Digital Health.

Comment #3

> *The background should be further expanded, for example, there are no mention about abstractive methods that create summaries without halluciantions, the authors just mention abstractive summaries "often produces fake contents that do not match the reference summary". A more complete review of previous studies should also include abstractive methods, such as implemented in the CliniText system and BT-45 for example, that did not produce any hallucinations.*

Thank you for the suggestion on the presentation of the related works. We modified the related works section and added some studies related to the tasks as we could. Please note that the study mentioned in the comment uses structured data as inputs. Meanwhile, our study targets unstructured data as the inputs.

Minnor comment #1

> *Typos: "In this study,105we adopt both of the two methods. in106Japanese"*

Thank you for the comment. We have corrected them in the revised manuscript.

Minnor comment #2

> *"However, it remains unclear how the summaries should be generated from the unstructured source" It’s unclear what the authors meant by unstructured source ? Images ? Free-text from other clinical experts ? Raw data ?*

Thank you for the suggestion. We have clarified the meaning of the “unstructured source” (Line 28).
